# Determinants of perinatal mortality among cohorts of pregnant women in three districts of North Showa zone, Oromia Region, Ethiopia: Community based nested case control study

**DOI:** 10.1186/s12889-018-5757-2

**Published:** 2018-07-18

**Authors:** Elias Merdassa Roro, Mitike Molla Sisay, Lynn M. Sibley

**Affiliations:** 1 School of Public Health, College of Health Sciences, Wollega University, Western, Ethiopia; 2 School of Public Health College of Health Sciences Addis Ababa University, Addis Ababa, Ethiopia; 3 School of Public Health College of Health Sciences, Post-Doctoral Fellow Emory University, Georgia, USA; 4 Nell Hodgson Woodruff School of Nursing, Rollins School of Public Health, Emory University, PI Maternal and Newborn Health in Ethiopia Partnership (MaNHEP), Georgia, USA

**Keywords:** Perinatal Mortality, Infant Mortality, Nested Case Control, Cohort, Verbal autopsy, MaNHEP, WHO

## Abstract

**Background:**

Statistics indicate that Ethiopia has made remarkable progress in reducing child mortality. It is however estimated that there is high rate of perinatal mortality although there is scarcity of data due to a lack of vital registration in the country. This study was conducted with the purpose of assessing the determinants and causes of perinatal mortality among babies born from cohorts of pregnant women in three selected districts of North Showa Zone, Oromia Region, Ethiopia. The study used community based data, which is believed to provide more representative and reliable information and also aimed to narrow the data gap on perinatal mortality.

**Methods:**

A community based nested case control study was conducted among 4438 (cohorts of) pregnant women. The cohort was followed up between March 2011 to December 2012 in three districts of Oromia region, Ethiopia, until delivery. The World Health Organization verbal autopsy questionnaire for neonatal death was used to collect data. A binary logistic regression model was used to identify determinants of perinatal mortality. Causes of deaths were assigned by a pediatrician and neonatologist. Cases are stillbirths and early neonatal death. Control are live births surviving of the perinatal period’

**Result:**

A total of 219 newborns (73 cases and 146 controls) were included in the analysis. Perinatal mortality rate was 16.5 per 1000 births. Mothers aged 35 years and above had a higher risk of losing their newborn babies to perinatal deaths than younger mothers [AOR 7.59, (95% CI, 1.91-30.10)]. Babies born to mothers who had a history of neonatal deaths were also more likely to die during the perinatal period than their counterparts [AOR 5.42, (95% CI, 2.27-12.96)]. Preterm births had a higher risk of perinatal death than term babies [AOR 8.58, (95% CI, 2.27-32.38)]. Similarly, male babies were at higher risk than female babies [AOR 5.47, (95% CI, 2.50-11.99)]. Multiple birth babies had a higher chance of dying within the perinatal period than single births [AOR 3.59, (95% CI, 1.20-10.79)]. Home delivery [AOR 0.23, (95% CI, 0.08-0.67)] was found to reduce perinatal deaths. Asphyxia, sepsis and chorioamnionitis were among the leading causes of perinatal deaths.

**Conclusion:**

This study reported a lower perinatal mortality rate. The main causes of perinatal death identified were often related to maternal factors. There is still a need for greater focus on these interrelated issues for further intervention.

## Background

Approximately eight million perinatal deaths are reported annually in the world, of which 40-60% is early neonatal mortality and almost all of which are found in developing countries ([1], Yaekob T, Mitike G: Assessment of pregnancy outcome with Emphasis on perinatal and neonatal Mortality in Dire Dawa town, Ethiopia, unpublished). In Africa, the perinatal mortality rate is more than six times higher than in developed countries [2, 3]. Many traditional societies do not name newborns until six weeks old, reflecting a sense of fatalism and cultural adaptation to high mortality [4, 5].

In Ethiopia, child mortality is still high and neonatal mortality contributes about 40% of all deaths [6]. Although there is scarcity of studies in perinatal mortality, a perinatal mortality audit performed at Jimma Hospital, Oromia region, Ethiopia, over a ten-year period, reported the highest perinatal mortality with a rate of 130 per 1000 live births [7]. The Ethiopian Demographic and Health Survey (EDHS 2011) reported the national perinatal mortality rate as 46 per 1000 pregnancies, showed a higher rate among women living in rural areas. The same study indicated a regional prevalence of 45 per 1000 pregnancies in the Oromia Region [8].

There appears to be no significant decline in mortality rate in the first week of life in the last 20-30 years. For example, in 1980, only 23% of all deaths were recorded in the first week of life; by the year 2000, this figure had unfortunately increased to an estimated 28% (3 million deaths). A review of studies conducted in Ethiopia between 1974 and 2013, both in hospitals and community based, did not show a reduction in perinatal mortality [9]. Stable trends of 90 and 40 per 1000 births were reported in hospitals and community settings, respectively over the past decades [9]. Another study from Hawassa University Referral Hospital, South Ethiopia, over the period 2008 -2010 reported adjusted perinatal mortality rate of 85 per 1000 total deliveries [10], which is more than two-fold higher than the estimated national perinatal mortality rate from EDHS 2011 [8]. Obstructed labor, malpresentation, preterm birth, antepartum hemorrhage and hypertensive disorders of pregnancy were independent predictors for perinatal deaths [10].

Although successive EDHS have shown a decline in child mortality, the perinatal mortality rate has remained high over the last decade. This study was, therefore, conducted to assess the main determinants of perinatal mortality in order to help inform health program managers and policy makers in their efforts to address this public health issue.

## Methods

### Study setting and nature of the cohort

This study was conducted in three districts of North Showa Zone of Oromia Regional State (Degem, Kuyu, and Wara Jarso). The zone is situated 100km from the capital, Addis Ababa. Most of the population in the study area were farmers. Around 84,558 women of reproductive age group (15-49) lived in the study area in 2010 [11], from which an open cohort of 4,438 pregnant women was established by mid-2010 in the three districts. The cohort was formed by Maternal and Newborn Health Partner in Ethiopia (MaNHEP) in collaboration with the Ethiopian Federal Ministry of Health, Emory University and Addis Ababa University [11].

Maternal and Newborn Health Partner in Ethiopia (MaNHEP) is a non-governmental organization, working to implement a health extension program by building the skills of frontline health workers and developing the systems needed to deliver quality maternal and newborn health care. It works in three districts of Oromia region, namely Degem, Kuyu, and Wara Jarso districts, and is led by Emory University, in collaboration with John Snow Research and Training Institute and Addis Ababa University, under the leadership of the Federal Ministry of Health.

**Fig. 1:**
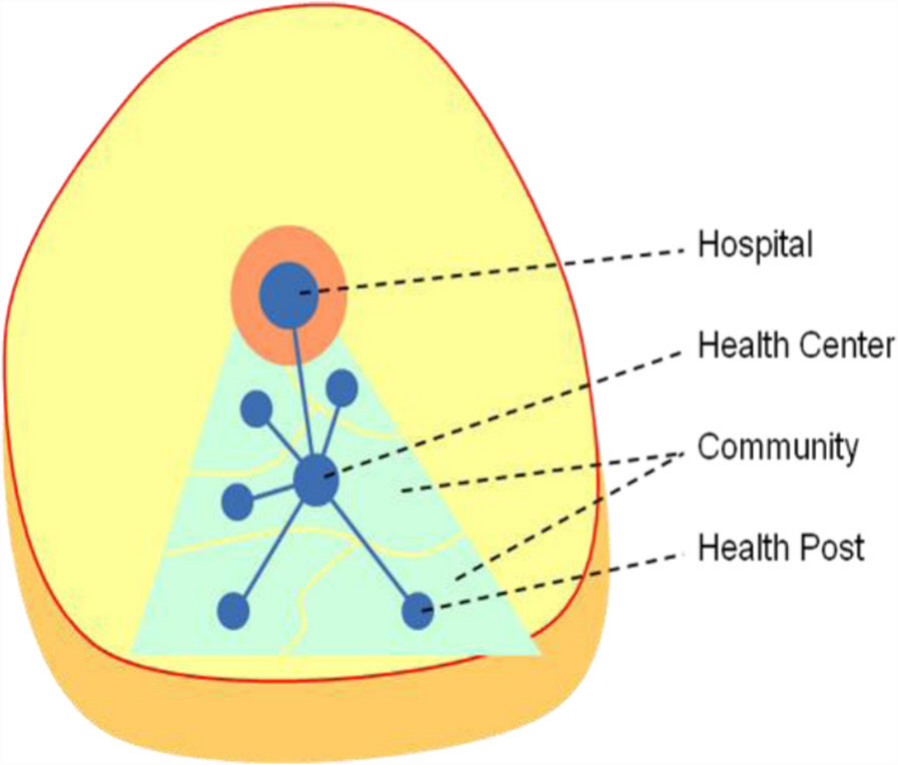
Study site components within One District. Taken from [11]

Twenty-seven kebeles (lower administrative units**)** were selected from the three districts, (8 kebeles from Degem district, 10 kebeles from Kuyu district and 9 kebeles from Wara Jarso district). The districts were among the most populous areas of the zone with a total population of 98, 2018 in Degem district, 126,546 in Kuyu district and 148,771in Wara Jarso district [12]. Kuyu hospital is the only hospital located in the study area. There were two health centers in each district and two health posts in each kebele (Fig. 1).

Pregnant women were enrolled in the cohort when they were around 28 weeks of gestation. The pregnant women were identified for the cohort by trained frontline health workers. Once in the cohort, every pregnant woman and their close family members (mothers, mothers-in-law, and husbands) received repeated training on care during pregnancy, labour, and delivery by the volunteers. Following delivery, they stayed in the cohort until they received postnatal care for the first 48 hours.

### Description of the intervention

**Fig. 2:**
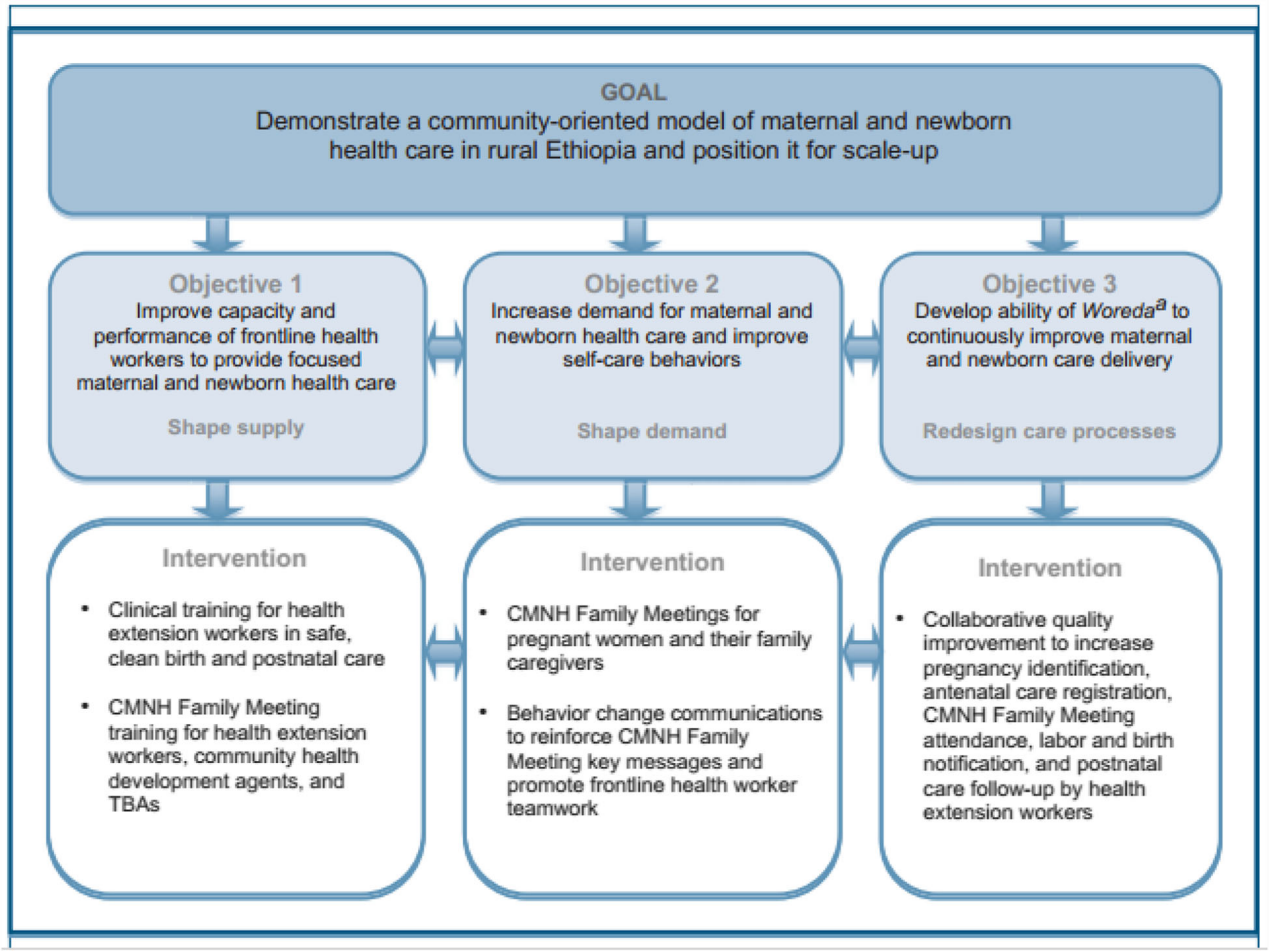
Goal, Objectives, and Key Interventions of the Maternal and Newborn Health in Ethiopia Partnership. Taken from Marge Koblinsky [37]

The project has developed an integrated program of maternal and newborn health training, quality improvement and behavioral change communications to ensure that the care reaches all women and newborn, in time, and every time. The main, objectives and key interventions of the project are described in Fig. 2.

### Study design, selection

A community based nested case control study design was used for this study. All perinatal deaths occurring between March 2011 and December 2012 were taken as cases. The controls were newborns from mothers included in the cohort who survived the perinatal period (first week). For every perinatal death, two controls were randomly selected by using a lottery method from the frame of the cohort. Controls were selected with a preference to those births that occurred in the same 'Gote' (smaller segment of a kebele), to avoid geographical disparities.

**Fig. 3:**
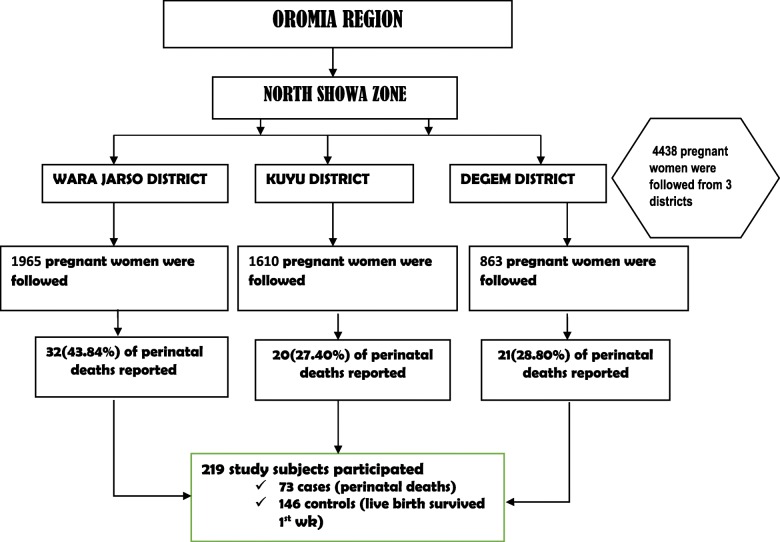
Schematic presentation of sampling procedure for study perinatal mortality in North Showa of Oromia region Ethiopia, 2012

Figure 3 shows a schematic of the sampling procedure for this study.

### Data collection instruments and procedures

Data was collected using the World Health Organization verbal autopsy questionnaire for neonatal death after adapting it to the local context [13]. The instrument contains about 12 sections, which includes the respondents’ basic demographic information, the deceased infants’ demographics, pregnancy history, delivery history and information about the family wealth. One experienced data collector was recruited for each district and trained for five days. Data from the controls was collected after gathering data from the cases using the sample frame which contained a list of all mothers who gave birth in the three districts. Mothers who lost their newborns were interviewed at least forty days after the death of the newborn in order to ensure their recovery from their bereavement. Face to face interview technique was used to gather the necessary information. The questionnaire was translated into the regional working language, Afaan Oromo, for easy comprehension. All data was collected by going to the house of each study respondent.

#### The cause of death assignment

The cause of deaths (COD) were assigned by a pediatrician and neonatologist. Each physician assigned the COD independently. The verbal autopsy (VA) data was reviewed and cause of death was assigned separately to every case. Finally, the physicians gave codes to the identified causes of deaths according to the ICD-10 coding system. A consensual diagnosis was reached by the two physicians following discussion. The classification style for cause of perinatal death presented in this manuscript was adapted to the Ethiopian context.

#### Statistical Method

Data were entered and cleaned using Epi Info version 3.5.1 and exported to SPSS version 20 statistical software for Windows for analysis. Univariate analysis was performed for each variable to measure the association between the dependent and individual independent variables. To control the effect of confounding variables, binary logistic regression models were applied using a hierarchical approach. As Binary Logistic Regression (BLR) was the main model used for multivariable analysis, it is used in this study to compare two groups i.e. ‘perinatal death’ as ‘cases’ and **‘**survivors of the perinatal period’ as ‘controls’**.** It is also preferred because the dependent variable had binary outcomes. The BLR model was applied using a hierarchical approach to see the effect of ‘socio-demographic variables’ on perinatal mortality independently as one group in one model and other ‘contextual variables’ in another model. Effect sizes were presented using crude and adjusted odds ratio at 95% CI.

### Variables

Perinatal death was the outcome variable. Pregnancy losses occurring after seven completed months of gestation (28 weeks) were considered as stillbirths. Deaths occurring to live births within the first seven days of life were considered as early neonatal death. Perinatal death is therefore defined as the sum of stillbirth and early neonatal death.

The independent variables under the socio-demographic factors were: the age of the mother, marital status, educational status, occupation, size of the household, and household financial situation. In addition, pregnancy, labour and delivery related variables such as gestational age (calculated from the last menstrual period), birth spacing, place of delivery, parity, history of neonatal death, history of abortion (both spontaneous and medically induced termination of pregnancy before the 28^th^ week of gestation) were included under demographic factors.

#### Household Wealth Index

Twelve dichotomous household asset variables were used to generate a wealth index using Principal Component Analysis (PCA) method. Household amenities used were mobile phone, radio, bed, kerosene lamp, plough, cash crops, tables, and chairs. According to the index, households were divided into quintiles ranging from the lowest to the highest.

## Results

### Mortality indexes and study population profile

During the study period, a follow up was made to 4438 pregnant women. 55 (1.2%) of these mothers were lost to follow-up and their pregnancy outcome are unknown. A total of 73 (1.7%) mothers lost their newborns during the perinatal period, of which 47 (63.4%) were early neonatal deaths, and 26 (35.6%) were stillbirths.

Overall perinatal mortality rate was 16.5 per 1000 total birth (95% CI: 13.0-20.6). The stillbirth rate was 5.9 per 1000 births (95% CI: 3.9-8.4). Early neonatal mortality rate was 10.2 per 1000 live births (95% CI, 3.9-8.4). The majority, 211 (96.35%), of the respondents were mothers, 7 (3.2%) were fathers and 1 (0.5%) was a sibling of the baby.

### Socio-demographic information of respondents

Mean age of mothers for both cases and controls was 27.01 years + 6.22 and 26.37 years + 4.70 respectively. Most of the mothers of controls were not able to read and write compared to mothers of cases during the perinatal period. Higher proportion of the family sizes of the cases were 35(47.9%) Three to Five, compared to the controls 77(52.7%) (Table 1).

**Table 1:** Socio-demographic and socio-economic characteristics of participants in North Showa zone, Oromia Region, Dec. 2012

Variables	Cases(n=73)	Controls(n=146)
Mothers age in years		
15-19	15(20.5%)	26(17.8%)
20-24	11(15.1%)	14(9.6%)
25-29	21(28.8%)	67(45.9%)
30-34	13(17.8%)	31(21.2%)
>35	13(17.8%)	8(5.5%)
Mean age + SD	27.01 + 6.22	26.37 + 4.70
Marital status		
Currently Married	66(90.4%)	141(96.6%)
Currently not married	7(9.6%)	5(3.4%)
Educational status of the mother		
Cannot read/write	52(71.2%)	122(83.6%)
Can read/write	21(28.8%)	24(16.4%)
Educational status of the father		
Cannot read/write	44(60.3%)	93(63.7%)
Can read/write	29(39.7%)	53(36.3%)
Occupation of the mother		
Farmer	62(84.9%)	137(93.8%)
Other	11(15.1%)	9(6.2%)
Family size		
Two	12(16.4%)	1(0.7 %)
Three to Five	35(47.9%)	77(52.7%)
Six and above	26(35.6%)	68(46.6%)
Sex of the new born		
Male	57(78.1%)	72(49.3%)
Female	16(21.9%)	74(50.7%)
Household Wealth index		
Lowest	8(28.6%)	21(33.9%)
Middle	12(42.9%)	20(32.3%)
Highest	8(28.6%)	21(33.9%)

### Health service utilization and related characteristics of respondents

In the study area, better health care service utilization patterns were observed in general compared to what other non-intervention sites the area. The majority of the respondents had a history of attending antenatal visits, 56 (76.7%) among cases and 121 (82.9%) among controls. Around 47 (64.4%) of the cases and 105 (71.9%) of the controls were vaccinated for tetanus toxoid, 51 (69.9%) of the cases and 119 (81.5%) of the controls had more than a two-year birth interval, and the majority of the births, 56 (76.7%) of the cases and 136 (93.2%) of the controls, were delivered at home (Table 2).

**Table 2:** Health service utilization and related characteristics of participants in North showa, Zone Oromia Region, Dec. 2012

Variables	Cases(n=73)	Controls(n=146)
Having ANC visit		
Yes	56(76.7%)	121(82.9%)
No	17(23.3%)	25(17.1%)
Gestational Age at first ANC		
First trimester	11(24.4%)	18(17.1%)
Second trimester	23(51.1%)	59(56.2%)
Third trimester	11(24.4%)	28(26.7%)
TT vaccination		
Yes	47(64.4%)	105(71.9%)
No	26(35.6%)	41(28.1%)
Dose of TT vaccination		
Single dose	6(12.8%)	20(19.0%)
Two to Three doses	35(74.5%)	73(69.5%)
Four to Five doses	6(12.8%)	12(11.4%)
Postnatal visit within 7 days		
Yes	34(46.6%)	72(49.3%)
No	39(53.4%)	74(50.7%)
Previous birth intervals		
Less than two years	15(20.5%)	18(12.3%)
Two years	7(9.6%)	9(6.2%)
More than two years	51(69.9%)	119(81.5%)
Walking distance from the nearest health facility		
< 1 hour	24(32.9%)	51(34.9%)
1-2 hours	42(57.5%)	91(62.3%)
> 2 hours	7(9.6%)	4(2.7%)
Place of delivery		
Home	56(76.7%)	136(93.2%)
Health Facility	17(23.3%)	10(6.8%)

### Pregnancy outcomes and related characteristics of respondents

A high number of the cases [50 (68.5%)] and the controls [106 (72.6%)] had had parity greater than three. Less than half, 28 (38.4%) of the cases and 21 (14.4%) of the controls had a history of neonatal mortality. Similarly, a minority, 11(15.1%) of the cases and 7 (4.8%) of the controls were preterm deliveries (Table 3).

**Table 3:** Pregnancy outcomes and related characteristics of participants in North Showa Zone, Oromia Region Dec. 2012

Variable	Cases(n=73)	Controls(n=146)
Birth Order		
First	15(20.5%)	20(13.7%)
Second	8(11.0%)	20(13.7%)
Third and higher	50(68.5%)	106(72.6%)
Parity		
One	15(20.5%)	20(13.7%)
Two to five	19(26.0%)	53(36.3%)
Six and above	39(53.4%)	73(50.0%)
History of abortion		
Yes	9(12.3%)	16(11.0%)
No	64(87.7%)	130(89.0%)
Previous History of neonatal mortality		
Yes	28(38.4%)	21(14.4%)
No	45(61.6%)	125(85.6%)
Type of birth		
Singleton	62(84.9%)	135(92.5%)
Multiple births	11(15.1%)	11(7.5%)
Preterm delivery		
Yes	11(15.1%)	7(4.8%)
No	62(84.9%)	139(95.2%)

### Determinant of perinatal mortality

To identify potential predictors of perinatal mortality, binary logistic regression models were run independently for socio-demographic factors and maternal-related factors.

It was found that, babies born to mothers aged 35 years and older were 7 times more likely to die during the perinatal period than those babies born to younger mothers [AOR 7.59, (95% CI, 1.913-30.102)]. Newborns from families with six or more family members had a higher chance of surviving the perinatal period than those babies born to families with five or fewer family members [AOR 0.083, (95% CI, 0.014-0.488)] (Table 4).

**Table 4:** Socio-demographic factors associated with perinatal mortality in North Showa Zone, Oromia Region, Dec. 2012

Variables	Cases(*n*=73)	Controls(*n*=146)	COR (95% CI)	AOR (95% CI)
Mother's age in years				
20-24	11(15.1%)	14(9.6%)	1	1
15-19	15(20.5%)	26(17.8%)	1.362(0.494-3.753)	0.428(0.100-1.828)
25-29	21(28.8%)	67(45.9%)	0.543(0.243-1.212)	0.802(0.329-1.958)
30-34	13(17.8%)	31(21.2%)	0.727(0.293-1.801)	2.081(0.616-7.022)
>35	13(17.8%)	8(5.5%)	2.817(0.951-8.345)	7.588(1.913-30.102) *
Marital status of the mother				
Currently Married	66(90.4%)	141(96.6%)	1	1
Currently not married	7(9.6%)	5(3.4%)	2.701(1.065-6.849)	3.604(0.940-13.812)
Educational status of the father				
Cannot read/write	52(71.2%)	122(83.6%)	0.865(0.485-1.541)	0.512(0.221-1.186)
Can read/write	21(28.8%)	24(16.4%)	1	1
Educational status of the mother				
Cannot read/write	44(60.3%)	93(63.7%)	0.487(0.249-0.952)	1.368(0.667-2.804)
Can read/write	29(39.7%)	53(36.3%)	1	1
Occupation of the mother				
Farmer	62(84.9%)	137(93.8%)	1	1
Other money earning jobs	11(15.1%)	9(6.2%)	2.701(1.065-6.849)	0.672(0.221-2.041)
Household Family size				
Two	12(16.4%)	1(0.7%%)	1	1
Three to Five	35(47.9%)	77(52.7%)	0.038(0.005-0.303)	0.263(0.062-1.112)
Six and above	26(35.6%)	68(46.6%)	0.032(0.004-0.257) *	0.083(0.014-0.488) *

*Significant at *P* value< 0.05, *COR* Crude Odds Ratio, *AOR* Adjusted Odds Ratio

On the other hand, babies born to mothers with a history of neonatal mortality had a higher risk of perinatal death compared to those babies born to mothers without a history of neonatal mortality [AOR 5.425, (95% CI, 2.271-12.960)]. Preterm births had a higher risk of death within the perinatal period than term babies [AOR 8.583, (95% CI, 2.275-32.386)]. Male babies were more likely to sustain perinatal death [AOR 5.478, (95% CI, 2.502-11.99 7)] than female babies. Multiple births were 3 times more likely to die within the perinatal period than singleton births [AOR 3.599, (95% CI, 1.200-10.798)]. Home delivery, however, was found to reduce the chance of perinatal death when compared to facility delivery [AOR 0.233, (95% CI, 0.081-0.6720)] (Table 5).

**Table 5:** Maternal and contextual factors associated with perinatal mortality in North Showa Zone Oromia Region, Dec. 2012

Variables	Cases(n=73)	Controls(n=146)	COR (95% CI)	AOR (95% CI)
Parity				
One	15(20.5%)	20(13.7%)	1	1
Two to five	19(26.0%)	53(36.3%)	0.478(0.204-1.119)	0.565 (0.120-2.660)
Six and above	39(53.4%)	73(50.0%)	0.712(0.328-1.545)	1.343(0.279-6.660)
Birth Order				
First	15(20.5%)	20(13.7%)	1	1
Second	8(11.0%)	20(13.7%)	0.533(0.185-1.537)	1.710 (0.236-12.406)
Third and higher	50(68.5%)	106(72.6%)	0.629(0.297-1.330)	1.001(0.183-5.485)
History of abortion				
Yes	9(12.3%)	16(11.0%)	1.143(0.479-2.727)	0.385 (1.119-1.247)
No	64(87.7%)	130(89.0%)	1	1
History of neonatal mortality				
Yes	28(38.4%)	21(14.4%)	3.704(1.913-7.169) *	5.425(2.271-12.960)*
No	45(61.6%)	125(85.6%)	1	1
Sex of the newborn				
Male	57(78.1%)	72(49.3%)	3.661(1.926-6.961)*	5.478(2.502-11.997)*
Female	16(21.9%)	74(50.7%)	1	1
Type of birth				
Singleton	62(84.9%)	135(92.5%)	1	1
Multiple births	11(15.1%)	11(7.5%)	2.177(0.896-5.293)	3.599(1.200-10.798)*
Preterm delivery				
Yes	11(15.1%)	7(4.8%)	3.523(1.304-9.517)*	8.583(2.275-32.386)*
No	62(84.9%)	139(95.2%)	1	1
Having ANC visit				
Yes	56(76.7%)	121(82.9%)	1	1
No	17(23.3%)	25(17.1%)	1.469(0.735-2.938)	0.555(0. 232-1.324)
Having TT vaccination				
Yes	47(64.4%)	105(71.9%)	1	1
No	26(35.6%)	41(28.1%)	1.417(0.778-2.581	1.618 (0. 744-3.519)
Birth spacing				
Less than two years	15(20.5%)	18(12.3%)	1.944(0.910-4.156)	2.935(0.437-19.732)
Two years	7(9.6%)	9(6.2%)	1.815(0.641-5.138)	0.850 (0. 219-3.306)
More than two years	51(69.9%)	119(81.5%)	1	1
Place of delivery				
Home	56(76.7%)	136(93.2%)	0.242(0.104-0.562)*	0.233(0.081-0.672)*
Health Facility	17(23.3%)	10(6.8%)	1	1
Walking Distance from health institution				
< 1 hour	24(32.9%)	51(34.9%)	1	1
1-2 hours	42(57.5%)	91(62.3%)	0.981(0.534-1.801)	0.336(0.068-1.653)
> 2 hours	7(9.6%)	4(2.7%)	3.719(0.993-13.932)	0.314(0.068-1.440)

* Significant at *P* value< 0.05, *COR* Crude Odds Ratio, *AOR* Adjusted Odds Ratio

### Physician reviewed probable causes of perinatal deaths

Asphyxia and sepsis were the major causes of early neonatal death (Table 6). The majority of the stillbirths resulted from chorioamnionitis and antepartum hemorrhage (Table 7).

**Table 6:** Physician assigned causes of Early Neonatal Death in North Showa Zone, Oromia Region Dec. 2012

S.NO	Probable Cause for Early neonatal deaths	Frequency
1	Birth Asphyxia	21(31.3%)
2	Bacterial Sepsis	17(25.4%)
3	Prematurity	2(3.0%)
4	Birth Injury	1(1.5%)
5	Tetanus	1(1.5%)
6	Pneumonia	1(1.5%)

**Table 7:** Physician assigned causes of still births in North Showa Zone, Oromia Region Dec. 2012

S.NO	Probable cause for still births	Frequency
1	Chorioamnionitis	8(11.9%)
2	Antepartum hemorrhage	6(9.0%)
3	Hypertensive disorder of pregnancy	5(7.5%)
4	Obstructed labor	2(3.0%)
5	Cardiac case (maternal pre-existing case)	1(1.5%)
6	Unknown causes	2(3.0%)

## Discussion

### Perinatal mortality rate

In this community based nested case control study, it was found that the perinatal mortality rate was 16.5 per 1000 total births. This finding was lower than previously reported in a review of community-based studies conducted in Ethiopia between 1974 and 2013, which gave a perinatal mortality rate of 40 per 1000 total births [9]. This observed reduction in this study may be related to the increase in Antenatal Care (ANC) coverage in the study area as a consequence of the MaNHEP intervention. A substantial increase in key health service coverage was observed during the intervention period in the study area compared to at baseline, for example, there was an increase in the ANC coverage in the study area because of the MaNHEP intervention [14]. A birth audit conducted in the study area reported that first ANC visit increased from 38% at baseline (November 2010), to 85% at end line (June 2012) [14]. The percentage of women receiving a postnatal care (PNC) visit within two days of delivery increased from 1% at baseline (November 2010) to 73% at end line (June 2012) [14]. This figure is seven times higher than the 2011 EDHS reported for postnatal care visit in the same study area [8]. In addition, the same study revealed 90%, 76% and 79% of births were conducted in a clean place, with clean helpers, and clean pregnant woman respectively even though most deliveries (89%) were at home [14].

Facility-based studies tend to overestimate the actual community-based perinatal mortality rate. For example, studies from Jimma and Hawasa referral hospitals from Ethiopia, reported perinatal mortality rates of 130 per 1000 live birth [7] and 85 per 1000 total delivery [10] respectively, while a review of hospital-based studies from Ethiopia, from 1974 to 2013, reported a perinatal mortality rate of 90 per 1000 total delivery [9]. These figures are much higher than the findings of the present study. This could be due to mothers presenting to hospital very late and with serious obstetric complications following referral from a primary health facility.

### Determinants of perinatal mortality

In this study, a statistically significant association was observed between mothers older than 35 years and perinatal mortality. This may be related to the fact that women who lose their newborns will try to catch up by having another child immediately after their loss [15]. This may increase the likelihood of perinatal mortality. A qualitative study conducted in the same area, as a part of this study, reported that women who lost their newborns decided to have another child as a replacement, this usually resulted in the subsequent death of the newborn [15]. This finding was in keeping with prospective cohort studies from rural Pakistan [16] and Tanzania [17].

A previous history of neonatal mortality was a strong determinant of perinatal mortality in this study. This finding is consistent with a population-based study from Missouri (USA), which showed that women with a previous infant mortality had an elevated risk of subsequent stillbirth, with the most profound increase observed among black women [18]. Mothers with a history of neonatal mortality during their first pregnancy were 5 times more likely to experience stillbirth in the second pregnancy [19, 20]. Other studies from Zimbabwe [19] and Sweden identified that previous stillbirth was one of the most eminent risk factors for perinatal mortality [21].

Preterm births were at an increased risk of perinatal mortality in this study. The overall proportion of preterm babies in this study was 8%, and 15% of all perinatal deaths were among the preterm babies. This finding is higher than in a study conducted in Addis Ababa in selected public health facilities, which reported 7.1% of perinatal deaths were in preterm births [22]. The difference seen may be attributed to home delivery and limited access to higher-level health care in this study, as it was conducted in a rural area. A study from Nepal also indicated similar findings [23]. Other studies from North East India and Tanzania revealed that the risk of perinatal death was high among preterm babies [17, 24], and a study from São Paulo concluded that preterm birth was the most significant risk factor for early neonatal mortality [25].

The study also found that male babies were at higher risk of perinatal death than their female counterparts, this was also found in an analysis of 22 years of cohort data from Butajira in rural Ethiopia [26] and in a similar study from the Netherlands [27]. Similarly, as in most developing countries, male child mortality exceeds female counterparts [5]. Infant and neonatal mortality were also higher in males, as found in community-based cohort studies from Zimbabwe [28], Australia and New Zealand [29], Pakistan [16] and Ethiopia [30]. This is also true among the whole population, presumably because of biological factors affecting the male infants [31, 32]. The excess of male deaths observed in this study may be as many/the majority were born to women who were at both extremes of age (<19 and >35) compared to female babies.

Multiple birth babies were more than four times at risk of death than singletons in this study. This is consistent with other studies and is a known risk factor for perinatal mortality? [30, 33].

### Causes of perinatal death

The leading cause of perinatal deaths identified in this study was birth asphyxia, which accounted for approximately 30% of early neonatal deaths. This may be due to the fact that most of the deaths (75%) occurred at home and were attended by unskilled birth attendants who lack the skills and equipment to help resuscitate asphyxiated babies at birth. This finding was substantiated by study findings from other developing countries like Pakistan [16, 34] and Bangladesh [35], where asphyxia ranked first among the leading causes of perinatal deaths.

Infections such as sepsis, tetanus, and pneumonia combined contributed to a similar number of deaths as birth asphyxia in this study. The high proportion of cases of sepsis may be related to unhygienic birthplace, unhygienic cord care at birth including cord cutting and tying materials, and cultural taboos related to taking babies out of the home even when the newborn is sick. In this study, tetanus made a relatively small contribution to perinatal death, which may be linked to better Tetanus Toxoid (TT) immunization at ANC due to national campaigns by the Federal Ministry of Health (FMOH), at different times in the study area. Studies elsewhere [13, 16, 34–36] reported similar findings although infection/sepsis were the most common cause of perinatal death. The discrepancy between the current study findings and other study reports may be due to differences in the demography of the study population, the health care system and perhaps, more importantly, the methodology used for assigning a cause of death. In this study, assignment of the cause of perinatal death was based on the local physician’s knowledge and familiarity with the expected diseases in the region so our data should be interpreted with caution.

Identified major causes of stillbirths were chorioamnionitis, antepartum hemorrhage, and hypertensive disorder during pregnancy, which are linked to maternal causes of death. This is similar to findings from India and Ethiopia [24, 7]. Information related to causes of death for stillbirths in this study may be less reliable since mothers themselves may not be able to remember what happened due to the stress and pain or their level of consciousness at the time of birth.

### Limitations of the study

It should be noted that this study was conducted in a rural area, where pregnancy notification is viewed as taboo. Though all necessary efforts were made to track every perinatal death in the study, there may be missed cases due to the harsh/complex nature of the study area. Data was collected based on the respondents’ answers to the questions; hence there may be misclassification of stillbirths and early neonatal deaths. Information related to causes of death for stillbirths in this study may be less reliable since mothers themselves were more likely to be unable to recall the history of signs and symptoms of the stillbirths at the time of birth. Gestational ages were calculated based on the last menstrual period. Birth weight data were not collected.

In spite of the limitations however, it is strongly believed that this study provides valuable information in the field of this study.

Note: This study demonstrates the status of perinatal mortality during the study period, which may have altered by the present time.

## Conclusions

This study reports a relatively lower perinatal mortality rate in a rural setting in Ethiopia. This may suggest that community based interventions could positively change the newborn outcomes. Maternal age greater than 35, having a history of neonatal mortality, multiple births, preterm births and male sex were independently identified as conferring an increased risk of perinatal mortality.

## Abbreviations

ANC: Ante-Natal Care
AOR: Adjusted Odds Ratio
CI: Confidence Interval
CODA: Cause of Death Assignment
EDHS: Ethiopian Demographic and Health Survey
MaNHEP: Maternal and New Born Health Initiative Partnership in Ethiopia
OR: Odds Ratio
PHCUs: Primary Health Care Units
SD: Standard Deviation
VA: Verbal Autopsy
WHO: World Health Organization

## References

[1] World Health Report. Make every mother and child count. World Health Organization Geneva, Switzerland. 2005;2005

[2] Haines A, Cassels A (2004). Can the millennium development goals be attained?. BMJ.

[3] United Nations Children’s Fund (2004). The Progress of Nations.

[4] Lawn JE, Zupan J, Begkoyian G, Knippenberg R (2006). Disease Control Priorities in Developing Countries. Newborn Survival.

[5] Lawn JE, Cousens S, Bhutta ZA, Darmstadt GL, Martines J, Paul V, Knippenberg R, Fogstadt H, Shetty P, Horton R (2004). Why are 4 million newborn babies dying each year?. Lancet.

[6] Central Statistical Agency [Ethiopia]. 2014. Ethiopia Mini Demographic and Health Survey 2014. Addis Ababa, Ethiopia

[7] Asheber G (2000). Perinatal mortality audit at Jimma Hospital, South- Western Ethiopia 1990-1999. Ethiopian Journal of Health Development.

[8] Central Statistical Agency [Ethiopia] and ICF International, 2012. Ethiopia Demographic and Health Survey (2011). Addis Ababa, Ethiopia, and Calverton, Maryland.

[9] Berhan Y., Berhan A. Perinatal mortality trends in Ethiopia: REVIEW. Ethiop J Health Sci. (Special Issue) September 2014; 29-49. 10.4314/ejhs.v24i0.4s PMC4249204 25489181

[10] Bayou G, Berhan Y (2012). Perinatal mortality and associated risk factors: a case-control study. Ethiop J Health Sci..

[11] Emory University. Report to Bill and Melinda Gates Foundation. Atlanta, USA. 2010:2010.

[12] Federal Democratic Republic of Ethiopia. Population Census Commission. Summary and statistical report of population and housing census 2007, Central Statistical Agency. Addis Ababa, Ethiopia. 2008;

[13] Darmstadt Gary L, Law Joy E, Costello A (2003). Perspectives; advancing the state of the world’s newborns. Bulletin of the World Health Organization.

[14] Maternal and New Born Health Ethiopia Partnership. MaNHEP Quarterly, Ensuring Care for Mothers and Babies in Time, Every Time. Spring: Or can be accessed at; 2012. https://www.manhep.org.

[15] Molla M, Yirgu R, Gebremariam A, Sibley L (2014). Qualitative Study of Attitudes and Values Surrounding Stillbirth and Neonatal Mortality Among Grandmothers, Mothers, and Unmarried Girls in Rural Amhara and OromiyaRegions, Ethiopia: Unheard Souls in the Backyard. J Midwifery Women's Health.

[16] Jehan I, Harris H, Salat S, Zeb A, Mobeen N, Pasha O, McClure Elizabeth M, Moore J, Wright L, Goldenberg R (2009). Neonatal mortality, risk factors, and causes: a prospective population-based cohort study in urban Pakistan. Bulletin of World Health Organization.

[17] Habib Abu N, Lie RT, Oneko O, Shao J, Bergsjø P, Daltveit AK (2008). Socio-demographic characteristics and perinatal mortality among singletons in North East Tanzania: a registry-based study. Journal Epidemiology Community Health.

[18] August E, Salihu H, Weldeselasse H, Biroscak B, Mbah A, Alio A. Infant mortality and subsequent risk of stillbirth: a retrospective cohort study. BJOG. 2011:1471–528. 10.1111/j.1471-0528.2011.03137.x 21933338

[19] Emmanuel T, Notion G, Sh G, Ch A, Mufuta T, Simukai Z. Determinants of perinatal mortality in Marondera district, Mashonaland East Province of Zimbabwe, 2009: a case-control study. Pan African Medical Journal. 2011;8(7) 10.4314/pamj.v8i1.71054 PMC3201615 22121416

[20] Salihu H, Duan J, Nabukera S, Mbah A, Alio A (2011). Younger maternal age (at the initiation of childbearing) and recurrent perinatal mortality. European Journal of Obstetrics & Gynecology and Reproductive Biology.

[21] Andersson T, Hogberg U, Bergstrom S (2000). Community based prevention of perinatal deaths; lessons from nineteenth-century Sweden. International Journal of Epidemiology.

[22] Tegegne Assefa B, Enquoselassie F, Yusuf L (2010). Birth to pregnancy interval and its effect on perinatal outcomes in Addis Ababa. Ethiopia; Ethiopian Journal of Reproductive Health.

[23] Shrestha S, Dangol Singh S, Shrestha M, Shrestha RPB (2010). The outcome of Preterm Babies and Associated Risk Factors in a Hospital. Journal of Nepal Medical Association.

[24] Mavalankar DV, Trivedi CR, Gray RH (2007). Levels and risk factors for perinatal mortality in Ahmedabad, India. Bulletin of the World Health Organization.

[25] Schoeps D, Furquim de Almeida M, Alencar Pereira G, França I Jr, et al. Risk factors for early neonatal mortality. Rev Saúde Pública. 2007;41(6) 10.1590/s0034-89102007000600017 18066471

[26] Berhane Y, Hogberg U (1999). Prolonged labor in rural Ethiopia: a community base study. African Journal of reproductive health.

[27] Garssen J, Anouschka van der M (2004). Perinatal mortality in the Netherlands Backgrounds of a worsening international ranking. Demographic Research.

[28] Sh F, Harlow S, Welch K, Gillespie B. Incidence of stillbirth and perinatal mortality and their associated factors among women delivering at Harare Maternity Hospital, Zimbabwe: a cross- sectional retrospective analysis. BMC Pregnancy and Childbirth. 2005; 10.1186/1471-2393-5-9 PMC1156907 15876345

[29] Evans N, Hutchinson J, Simpson MJ, Donoghue D, Darlow B, Henderson-Smart D (2007). Prenatal predictors of mortality in very preterm infants cared for in the Australian and New Zealand Neonatal Network. Arch Dis Child Fetal Neonatal Ed.

[30] Assefa M, Drewett R, Tessema F (2002). A birth cohort study in South-West Ethiopia to identify factors associated with infant mortality that is amenable to intervention. Ethiopian Journal Health Development.

[31] Child Health Research Project Special Report (1999). Reducing Perinatal and Neonatal Mortality. Baltimore MD, Johns Hopkins University Baltimore, Maryland.

[32] Mondal Islam N, Hossain K, Ali K (2009). Factors Influencing Infant and Child Mortality: A Case Study of Rajshahi District, Bangladesh. Journal of Human Ecology.

[33] Hama A, Meda N, Zabsonré E, Sommerfelt H, Cousens S, Tylleskär T (2010). Perinatal mortality in Rural Burkina Faso: a prospective Community based cohort study. BMC Pregnancy and Childbirth.

[34] Zulfiqar Ahmed B., Zahid Ali M., Shujaat Z Farukkh R Adnan H. Verbal Autopsy Survey of Perinatal Mortality in Rural Pakistan; Child Health and Nutrition Research Initiative (CHNRI),2007.

[35] Chowdhury Rahanm H, Thompson S, Ali M, Alam N, Md Y, Streatfield Peter K (2010). Causes of Neonatal Deaths in a Rural Subdistrict of Bangladesh: Implications for Intervention. Journal of Health and Population Nutrition..

[36] Zupan J (2003). Perinatal Mortality and Morbidity in Developing Countries. Med Trop.

[37] M Koblinsky. Reducing Maternal and Perinatal Mortality Through a Community Collaborative Approach: Introduction to a Special Issue on the Maternal and Newborn Health in Ethiopia Partnership (MaNHEP). Journal of Midwifery & Women’s Health (Special Issue). January/February 2014; 59(1) Can be accessed at https://www.jmwh.org. 10.1111/jmwh.12174 24588910

